# Interfacial microscopic examination and chemical analysis of resin-dentin interface of self-adhering flowable resin composite

**DOI:** 10.12688/f1000research.12306.4

**Published:** 2018-10-23

**Authors:** Tamer M. Hamdy

**Affiliations:** 1 Restorative and Dental Materials Department, National Research Centre (NRC), El Bohouth St., 12622 Dokki, Giza, Egypt; 2 Dr. Tamer Hamdy Dental Clinic, Giza, Egypt

**Keywords:** Self-adhering, total-etch, bonding system, resin composite, gap distance, resin-dentin interface

## Abstract

**Background:** The newly introduced self-adhering flowable resin-composites decrease the required time for application by incorporation of an acidic adhesive monomer, thus reducing the number of steps, but its bonding is still uncertain. The aim of this study was to evaluate the interfacial microscopic examination and chemical analysis at the resin-dentin interface of a self-adhering flowable resin composite (Vertise™Flow Self-Adhering Flowable Composite, Kerr Dental, USA) versus a total-etch (Te-Econom Plus) resin composite, using an etching agent (Eco-Etch gel) and bonding agent (Single Bond Universal).

**Methods:** Sixteen freshly extracted sound human posterior teeth were used. The teeth were randomly divided into two groups: 8 specimens per type of composite. Standard-shaped class V cavities were prepared on the buccal surface. One group was restored by Te-Econom Plus resin composite by total-etch technique using Eco-Etch gel, which was applied to dentine for 15 seconds, followed by rinsing, drying and bonding agent application (Single Bond Universal). The other group restored directly with self-adhering resin composite (Vertise-Flow) without application of etch or bond. Curing was done for 20 seconds using a light emitting diode light curing unit. Evaluation of the resin-dentin interface was done microscopically by examination of marginal gap distance in μm using scanning electron microscope (SEM), and chemical analysis of silver particles was observed using SEM with energy-dispersive X-ray spectrometry after 24 hours of specimen storage in ammoniacal silver nitrate.

**Results:** Regarding marginal gap distance (µm) and silver atomic % mean values, teeth restored with self-adhering resin composite (Vertise-Flow) showed significantly higher mean values than the multi-step etch and rinse resin composite group (5.2 *vs *0; 12.2 *vs *8.2, respectively).

**Conclusions:** Resin-dentin bonding using total-etch resin composite technique was more effective than self-adhering flowable resin composite (Vertise-Flow) regarding marginal gap formation and penetration of silver particles. Further studies for bond strength could be performed.

## Introduction

Adhesive dentistry has seen a paradigm shift from the invasive to be minimally invasive, due to a revolution in bonding systems. There are great demands for simplified restorative materials. A new self-adhering flowable resin composite (Vertise™ Flow Self-Adhering Flowable Composite, Kerr Dental, USA), was recently introduced onto the market. Bonding of flowable composites to tooth structure is achieved by incorporation of an acidic adhesive monomer into the material ^1^. It is still a big challenge to seal the resin-dentin interface ^2, 3^.

The total-etch (etch and rinse) technique is a widely accepted technique to improve bonding of dental resins to tooth structure ^2^. The dentin bonding mechanism is based on the micro-mechanical interlocking of the infiltrated resin monomers into porosities created in demineralized inorganic part ^4^. Debonding of restorations may arise from gap-formation at the resin-dentin interface and hence recurrent caries, discoloration and tooth pain may follow ^5^. Thus sufficient marginal seal should be obtained. Recently, an innovative self-adhesive and flowable resin composite was developed. These materials are claimed to eliminate the need for a separate step of bond-application, finally simplifying the restorative procedure. Therefore, the aim of this study was to evaluate the sealing performance of this new material.

## Methods

### Preparation of specimens

After attaining written informed consent from each patient to use their extracted teeth in research, sixteen sound human molar teeth were extracted in a private dental clinic (Dr. Tamer Hamdy Dental Clinic), which were randomly divided into two groups (eight specimens per group). Standard-shaped class V cavities (3 mm width, 3 mm length, 2 mm of depth) were prepared in the teeth using a #169L carbide bur (KG Sorensen, Brazil) on the buccal surface. One group’s (Group A) cavities were filled with Te-Econom Plus ^®^ (Ivoclar Vivadent, Africa) resin composite after etching and bond application. The etching agent, Eco-Etch gel (Ivoclar Vivadent), was applied to dentine for 15 seconds, followed by rinsing and drying. After rinsing, a bonding agent (Single Bond Universal, 3M ESPE, USA) was applied to teeth for 20 seconds, afterwards the teeth were air-dried for 5 seconds and light-cured for 10 seconds. Finally, the Te-Econom Plus resin composite was applied. The other group’s (Group B) cavities were filled with self-adhering resin composite (Vertise™Flow Self-Adhering Flowable Composite, Kerr Dental, USA), which was applied without etch or bond. Curing was done for 20 seconds using a light emitting diode (LED) light curing unit (Satelec, Acteon, France).

### Interfacial microscopic examination

All teeth were stored in distilled water for 24 hours at 37°C. Subsequently, the specimens were vertically sectioned with a diamond saw (Isomet, Buehler Ltd., USA) under water lubrication into approximately 1mm thick slab composed of tooth structure bonded to resin composite. These were examined for marginal gap distance in μm using scanning electron microscope (SEM; Model Quanta 250 FEG; FEI, Thermo Fisher Scientific, USA): accelerating voltage 30 K.V., magnification 14x up to 1000000 and resolution for Gun.1n, to ensure high brightness and resolution at low accelerating voltage.

### Chemical analysis of the interface

Specimen slabs were placed in freshly prepared 50 weight % ammoniacal silver nitrate solution for an additional 24 hours at 37°C in the dark. Ammoniacal silver nitrate solution (pH=9.5) was prepared according to *Tay et al. (2002)* ^6^. After 24 hours of storage in silver nitrate solution, the silver impregnated specimens were rinsed thoroughly in distilled water and placed in photo-developing solution for 8 hours under a fluorescent light (200 Watt) ^3^.

The specimens were then observed under environmental SEM Model Quanta 250 FEG attached with energy-dispersive X-ray (EDX; Inspect S 50, FEI, Netherlands): accelerating voltage 30 K.V., magnification 4000x and resolution for Gun.1n. The Blackscattered backscattered electron mode was used for elemental analysis of the atomic silver %.

### Statistical analysis

Numerical data were explored for normality using Kolmogorov-Smirnov and Shapiro-Wilk tests, followed by Student’s t-test to compare between both groups. The significance level was set at P ≤ 0.05. Statistical analysis was performed with IBM ^®^SPSS ^®^ Version 20 for Windows (SPSS Inc., IBM Corporation, USA).

## Results

**Figure 1.**
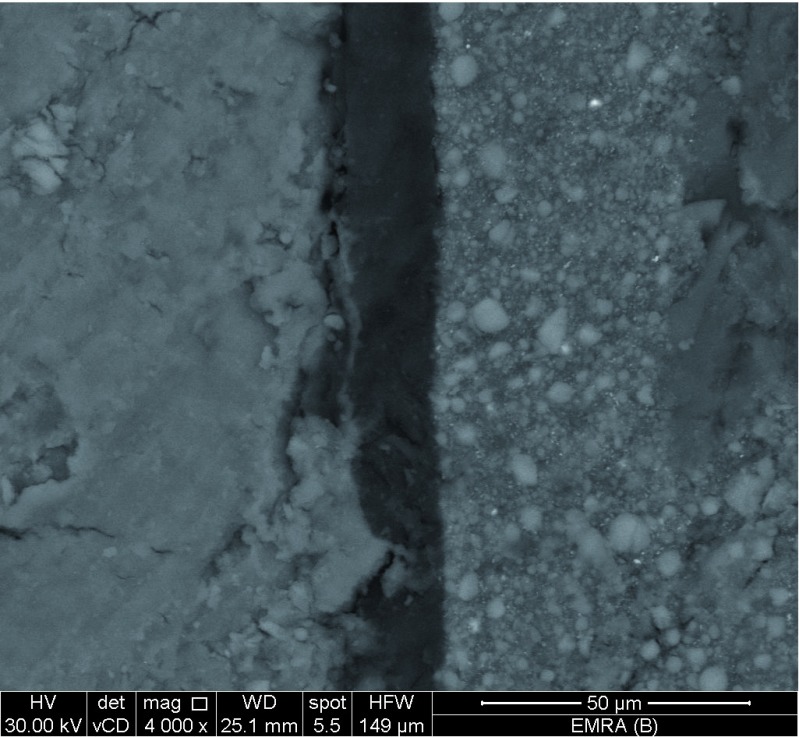
Selected scanning electron microscopy shows absence of gap formation in dentin for teeth treated with total-etch technique. Image representative of 8 teeth.

**Figure 2.**
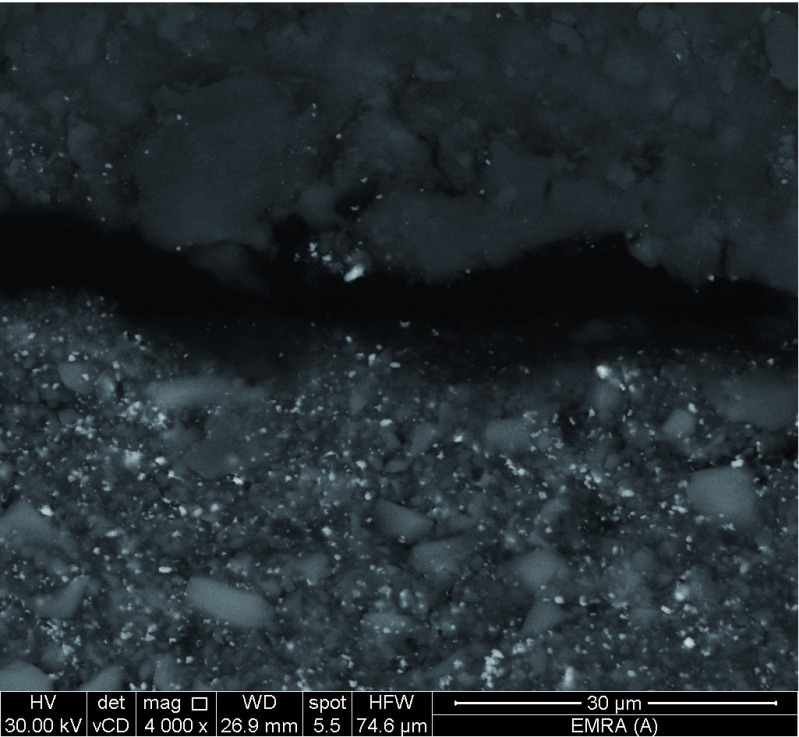
Selected scanning electron microscopy shows presence of gap formation in dentin for teeth treated with a self-adhering resin composite. Image representative of 8 teeth.

Regarding marginal gap formation, Group A showed a significantly lower mean gap distance values than Group B (p<0.001), as shown in Table 1 and Figures 1 and 2.

**Table 1.** Marginal gap formation (µm) between groups of teeth restored with different composites. Group A, treated with total-etch technique; Group B, treated with self-adhering resin composite. Mean and standard deviation (SD) values and results of Student’s t-test for the comparison between gap distances are shown (n=8/group).

Group A		Group B		P-value
Mean	SD	Mean	SD	
0.0	0.0	5.2	0.3	<0.001 ^*^

*: Significant at P ≤ 0.05

**Figure 3.**
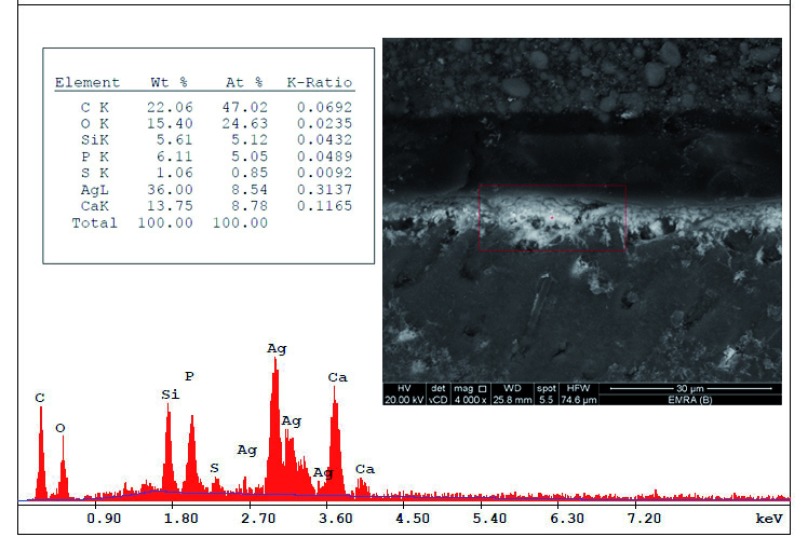
Selected scanning electron microscopy/energy-dispersive X-ray analysis at the resin-superficial dentin interfaces in teeth treated with total-etch technique. Image representative of 8 teeth.

**Figure 4.**
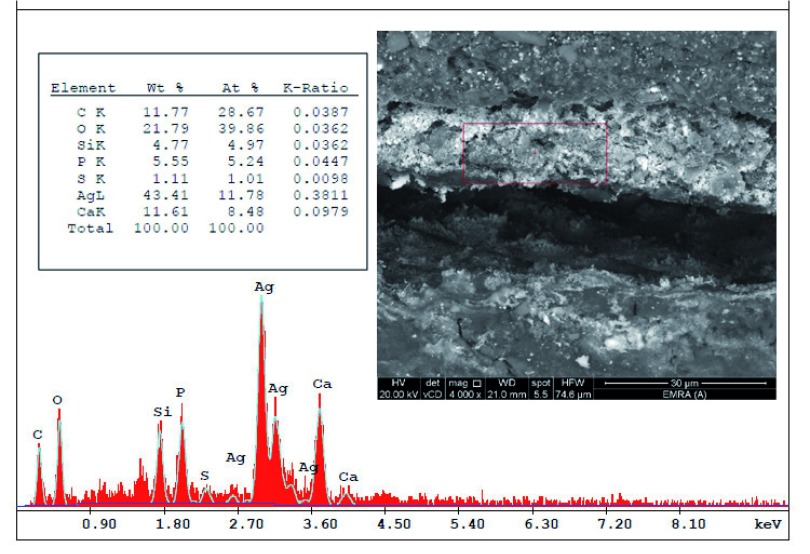
Selected scanning electron microscopy/energy-dispersive X-ray analysis at the resin-superficial dentin interface in teeth treated with self-adhering resin composite. Image representative of 8 teeth.

The SEM with EDX analysis results revealed significantly lower mean silver atomic % values for Group A compared to Group B (p< 0.001). A selected SEM/EDX analysis is shown in Table 2, and Figures 3 and 4.

**Table 2.** Chemical analysis of the interface between groups of teeth restored with self-adhering resin composite. Group A, treated with total-etch technique; Group B, treated with self-adhering resin composite. Mean and standard deviation (SD) values and results of Student’s t-test for the comparison between silver atomic % values are shown (n=8/group).

Group A		Group B		*P*-value
Mean	SD	Mean	SD	
8.2	0.4	12.2	0.7	<0.001 ^*^

*: Significant at P ≤ 0.05

Raw values for silver atomic % in teeth treated with total-etch technique (Group A) and self-adhering resin composite (Group B) (n=8/group/method) Click here for additional data file. Copyright: © 2018 Hamdy TM 2018 Data associated with the article are available under the terms of the Creative Commons Zero "No rights reserved" data waiver (CC0 1.0 Public domain dedication).

Scanning electron microscopy (SEM) showing gap formation (raw values included on the images) and SEM/energy-dispersive X-ray analysis (EDX) at the resin-superficial dentin interface in teeth treated with total-etch technique (Group A) and self-adhering resin composite (Group B) (n=8/group/method) Click here for additional data file. Copyright: © 2018 Hamdy TM 2018 Data associated with the article are available under the terms of the Creative Commons Zero "No rights reserved" data waiver (CC0 1.0 Public domain dedication).

## Discussion

A proper marginal seal of restoration is essential to improve the durability of resin composite/bonding systems ^7^. Most of the clinical studies assessing the performance of an adhesive system use class V cavities ^8^. EDX analysis permits identification of silver particles, thus giving an indication about the chemical analysis of the interface ^9^.

Our results revealed better sealing ability of composites treated with multi-step etch and rinse technique, presenting lower marginal gap formation and lower penetration of silver particles compared to Vertise-Flow. The presence of exposed collagen fibers could increase micromechanical interlocking of the bonding agent within the dentin surface ^10^. The poorer sealing of Vertise-Flow may be due to included adhesive monomer, the glycerol that etches instead of bonds to hydroxyapatite ^11^.

## Conclusions

Total-etch resin composite technique was more effective regarding marginal gap formation and penetration of silver particles as compared to a flowable resin composite (Vertise-Flow). Further studies on bond strength should be undertaken.

## Data availability

The data referenced by this article are under copyright with the following copyright statement: Copyright: © 2018 Hamdy TM

Data associated with the article are available under the terms of the Creative Commons Zero "No rights reserved" data waiver (CC0 1.0 Public domain dedication). 

Dataset 1: Raw values for silver atomic % in teeth treated with total-etch technique (Group A) and self-adhering resin composite (Group B) (n=8/group/method). doi, 10.5256/f1000research.12306.d177061 ^12^

Dataset 2: Scanning electron microscopy (SEM) showing gap formation (raw values included on the images) and SEM/energy-dispersive X-ray analysis (EDX) at the resin-superficial dentin interface in teeth treated with total-etch technique (Group A) and self-adhering resin composite (Group B) (n=8/group/method). doi, 10.5256/f1000research.12306.d177062 ^13^
