# Supplementary material for: Interfacial microscopic examination and chemical analysis of resin-dentin interface of self-adhering flowable resin composite
Source: F1000Res. 2018 Oct 23;6:1688. Originally published 2017 Sep 14. [Version 4] doi: 10.12688/f1000research.12306.4 (PMC5649117; doi:10.12688/f1000research.12306.4)
Supplement: Peer review history

# Peer review: Referee response for version 3

**DOI:** 10.5256/f1000research.14079.r27183

**Published:** 2017-10-26

**Authors:** Rania El-Saady Badawy

**Affiliations:** 1 Department of Dental Materials, Faculty of Dentistry, Suez Canal University, Ismailia, Egypt

The author has corrected all points required satisfactorily.

I have read this submission. I believe that I have an appropriate level of expertise to confirm that it is of an acceptable scientific standard.

# Peer review: Referee response for version 3

**DOI:** 10.5256/f1000research.14079.r27182

**Published:** 2017-10-25

**Authors:** Nadia A. Badr

**Affiliations:** 1 Department of Dental Biomaterials, Faculty of Oral and Dental Medicine, Cairo University, Cairo, Egypt

The article still lacks the name of company and country of production of the investigated materials (Vertise-Flow) in section of methodology.

I have read this submission. I believe that I have an appropriate level of expertise to confirm that it is of an acceptable scientific standard.

# Peer review: Referee response for version 2

**DOI:** 10.5256/f1000research.13966.r27027

**Published:** 2017-10-18

**Authors:** Rania El-Saady Badawy

**Affiliations:** 1 Department of Dental Materials, Faculty of Dentistry, Suez Canal University, Ismailia, Egypt

The following points need to be corrected, otherwise it is approved

- is based on the micro-mechanical interlocking of the infiltrated resin monomers into porosities created by demineralized inorganic part of dentin 4.
**Comment:** created in demineralized in organic part....
- Chemical analysis of the interface Specimens slabs were then placed in freshly prepared 50 weight/volume % ammoniacal silver nitrate solution for an additional 24 hours at 37°C in the dark.
**Comment:** Specim en slab s were placed.......
- lower mean gap distance values than Group B (p<0.001), as shown in Table 1 and Figure 1 and Figure 2.
**Comment:** and Figur es 1 and 2
- The SEM with EDX analysis results revealed that Group A showed significantly lower mean silver atomic % values than Group B (p<0.001), as shown in Table 2 A. Selected SEM/EDX analysis is shown in Figure 3 and Figure 4.
**Comment:** The SEM with EDX analysis results revealed significantly lower mean silver atomic % values for Group A compared to Group B (p< 0.001). A selected SEM/EDX analysis is shown in Figu res 3 and 4.
- A proper marginal sealing is essential to improve the durability of resin composite/bonding systems 7
**Comment:** ****A proper marginal se al is essential
- This may be attributed to the fact that phosphoric acid included in acid-etch step demineralized the smear layer, exposing the collagen fibers of superficially demineralized dentin. These could increase micromechanical interlocking of the bonding agent within the dentin surface 10
**Comment:** The presence of exposed collagen fibers could increase....

I have read this submission. I believe that I have an appropriate level of expertise to confirm that it is of an acceptable scientific standard.

# Response

**Published:** 2017-10-18

**Authors:** Tamer Hamdy

**Affiliations:** National Research Centre, Egypt, Egypt

Thanks for your concern, corrections done.

# Peer review: Referee response for version 1

**DOI:** 10.5256/f1000research.13321.r26333

**Published:** 2017-10-11

**Authors:** Nadia A. Badr

**Affiliations:** 1 Department of Dental Biomaterials, Faculty of Oral and Dental Medicine, Cairo University, Cairo, Egypt

The scientific idea of this article to evaluate the adaptability of resin/dentine interface of the most commonly used direct aesthetic restorative material is superb.

 The article lacks the name of the company & country of the main investigated material (flowable composite, Vertise-Flow).

 Despite the used method is well known for detection of nanoleakage of restorative materials, it is smartly employed for assessment of adaptability of composite at resin/dentine interface.

 The suggestions is logical but it is preferable to be separate item not involved in context of conclusion section.

**Summary:**

1. The article is presented clearly & accurately
2. The study design is appropriate
3. Sufficient details were provided except the name of the company & country of the investigated material (Vertise-Flow)
4. The used  method for detection of marginal gap is smartly employed
5. The data are reproducible
6. And the unique conclusion is obvious
7. The suggestion is promising for further researches

I have read this submission. I believe that I have an appropriate level of expertise to confirm that it is of an acceptable scientific standard.

# Response

**Published:** 2017-10-18

**Authors:** Tamer Hamdy

**Affiliations:** National Research Centre, Egypt, Egypt

Many thanks for your valuable comments, I do the required corrections, belong to "the suggestions is logical but it is preferable to be separate item not involved in context of conclusion section" I do that according to editorial request according to the journal role

# Peer review: Referee response for version 1

**DOI:** 10.5256/f1000research.13321.r26488

**Published:** 2017-10-06

**Authors:** Rania El-Saady Badawy

**Affiliations:** 1 Department of Dental Materials, Faculty of Dentistry, Suez Canal University, Ismailia, Egypt

After reviewing the entire manuscript, several modifications were added to different parts of the manuscript.

 For the Introduction part, it shortly covered the idea of investigated resin composites describing the aim of the study.

 Materials and Methods described steps and method of slab preparation (missing a few details).

 Results were objectively presented.

 The results were vaguely discussed.

 This manuscript is approved for publication after elaborating the changes required.

 Specific Comments:

  

- *Bonding is achieved by incorporation of an acidic adhesive monomer into the flowable composites ^1^*.

Comment: Bonding of flowable composites to tooth structure is achieved by incorporation of an acidic adhesive monomer into the material.

  

- *It is still a big challenge to seal the resin-dentin interface, due to the heterogeneous nature of dentin, the wet tubular structure, composition and surface morphology and or improperly designed adhesives ^2,3^* 

Comment: Too long sentence

  

- *The total-etch (etch and rinse) technique is a widely accepted technique to improve* *bonding of dental resins ^2^.*

Comment: improve bonding of dental resins to tooth structure

  

- *...is based on the micro-mechanical interlocking of the infiltrated resin monomers into porosities created by demineralized inorganic part of dentin ^4^*.

Comment: created in demineralized organic part....

  

- *Restoration debonding may arise from gap formation at the resin-dentin interface and hence recurrent caries, discoloration and tooth pain ^5^*

Comment: Debonding of restorations may arise from gap-formation ..........., discoloration and tooth pain may follow

  

- *These materials are claimed to eliminate the need for a separate bonding application step, thus simplifying the restorative procedure*

Comment: to eliminate the need for a separte step of bond-application, finally simplifying the restorative procedure

  

- *Thus, the aim of this study was to evaluate the sealing performance of this new material*

Comment: Therefore, the aim of.....

  

- *Preparation of specimens. After attaining written informed consent from each patient to use their extracted teeth in research*

Comment: Why should we get a patients approval to work on extracted teeth?? You should better start with: Sixteen sound human molar teeth.......

  

- . *..sixteen sound human posterior molar teeth were extracted in a private*

 Comment: Only say human molar teeth- it is known that molars are posterior

  

- *(eight specimens per group). Standard-shaped class V cavities (3x3 mm, 2 mm of depth) were prepared*

Comment: Dimensions of the cavity are unclear

  

- *One group’s (Group A) cavities were filled with Te-Econom Plus® (Ivoclar Vivadent, Africa) resin composite after etching and bonding application.*

Comment: and bond application

  

- *A bonding agent (Single Bond Universal, 3M ESPE, USA) was applied to the teeth for 20 Seconds*

Comment: After rinsing, a bonding agent ......... was applied to the teeth for 20 sec

   

- *...teeth for 20 seconds, then air-dried for 5 seconds, then light-cured for 10 seconds.*

   Comment: was applied to teeth for 20 seconds, afterwards the teeth were air-dried for 5 sec and light-cured for 10 sec

  

- *...under water lubrication into approximately 1mm thick slabs.*

Comment:  Slab of what??? Please explain specific composition the slab. Please mention that it is a slab composed of tooth structure bonded to resin composite of any type!!!

  

- *Chemical analysis of the interface. Specimens slab were then placed in freshly prepared 50 weight/volume % ammoniacal silver nitrate solution for an additional 24 hours at 37°C in the dark*.

Comment: Specimen slabs were placed.......weight or volume percent????

  

- *Ammoniacal silver nitrate solution (pH=9.5) was prepared according to the protocol of Tay et al. (2002) ^6^*.

 Comment: prepared according to Tay *et al*

  

- *After 24 hours of storage in ammoniacal silver nitrate, the silver impregnated specimens were then rinsed thoroughly in distilled water and placed in photo-developing*

  Comment: storage in silver nitrate solution, the silver impregnated specimens were rinsed. Thoroughly

  

- *The specimens were then observed under environmental SEM Model Quanta 250 FEG attached with energy-dispersive X-ray (EDX; Inspect S 50, FEI, Netherlands): accelerating voltage 30 K.V., magnification 4000x and resolution for Gun.1n.*

Comment: he treated specimens were observed.

- *Backscattered electron mode was used for elemental analysis of the atomic silver %.*

Comment: The Blackscattered ......

- *...followed by Student’s t-test to compare between the two groups.*

Comment: to compare between both groups

  

- *lower mean gap distance values than Group B (p<0.001), as shown in Table 1 and Figure 1 and Figure 2.*

Comment: and Figures 1 and 2

  

- *The SEM with EDX analysis results revealed that Group A showed significantly lower mean silver atomic % values than Group B (p<0.001), as shown in Table 2 A. Selected SEM/EDX analysis is shown in Figure 3 and Figure 4.*

  Comment: revealed significantly lower mean silver atomic % values for Group A compared to Group B (p< 0.001) A selected..... in Figures 3 and 4.

  

- ***Table 1. Marginal gap formation (μm) between groups of teeth treated with different composites***

Comment: groups of teeth restored with different...

  

- ***Figure 1. Selected scanning electron microscopy shows absence of gap formation in dentin in teeth treated with total etch technique.***

Comment: in dentin for teeth treated with....

  

- ***Figure 2. Selected scanning electron microscopy shows presence of gap formation in dentin in teeth treated with self-adhering resin composite.**      *

Comment:  in dentin for teeth........ with a self-adhering ....

  

- ***Table 2. Chemical analysis of the interface between groups of teeth treated with different composites.***
*Group A, treated with total-etch technique; Group B, treated with self-adhering resin composite.*

Comment:  restored with self-adhering resin composite

  

- ***Dataset 1. Raw values for silver atomic % in teeth treated with total-etch technique***
***(Group A) and self-adhering resin composite (Group B) (n=8/group/method)***

Comment: Group (A), …

  

- ***Dataset 2. Scanning electron microscopy (SEM) showing gap formation (raw values included on the images) and SEM/energy dispersive X-ray analysis (EDX) at the resin-superficial dentin interface in teeth treated with total-etch technique (Group A) and self-adhering resin composite (Group B) (n=8/group/method)***

Comment: Group (A), …

  

- *A proper marginal sealing is essential to improve the durability of resin composite/bonding systems ^7^*

 Comment: sealing of what???

  

- *From the present results, it was obvious that the multi-step etch and rinse technique provides better sealing regarding*

 Comment: Our results revealed better sealing ability of composites treated with multi-step etch and rinse technique, presenting lower marginal gap formation and lower penetration of silver particles compared to Vertise-Flow.

  

- *This may be attributed to the fact that phosphoric acid included in acid-etch step demineralized the smear layer, exposing the collagen fibers of superficially demineralized dentin.*
* These could increase micromechanical interlocking of the bonding agent within the dentin surface ^10^*

Comment: Presence of exposed collagen fibers could increase....

  

- *The poorer sealing of Vertise-Flow may be due to the included adhesive monomer, which is called glycerol phosphate dimethacrylate “GPDM”, that etches instead of bonds to hydroxyapatite ^11^.*

Comment: included adhesive monomer, the glycerol.........

  

- *Further studies for bond strength could be performed. It is important to emphasize that this study ignored the effect of oral condition,*

Comment: Further studies on bond strength should be undertaken.

I have read this submission. I believe that I have an appropriate level of expertise to confirm that it is of an acceptable scientific standard, however I have significant reservations, as outlined above.

# Response

**Published:** 2017-10-06

**Authors:** Tamer Hamdy

**Affiliations:** National Research Centre, Egypt, Egypt

Thanks for your valuable revision. Corrections done

Belong to your comment

Comment: Why should we get a patients approval to work on extracted teeth?? You should better start with: Sixteen sound human molar teeth......

It was done according to journal editor revision due to the rule of the journal.
